# Additional benefit of induced pluripotent stem cell-derived mesenchymal stem cell therapy on sepsis syndrome-associated acute kidney injury in rat treated with antibiotic

**DOI:** 10.1186/s13287-021-02582-5

**Published:** 2021-10-07

**Authors:** Chih-Chao Yang, Pei‐Hsun Sung, Chih-Hung Chen, John Y. Chiang, Pei-Lin Shao, Shun-Cheng Wu, Hon‐Kan Yip

**Affiliations:** 1 Division of Nephrology, Department of Internal Medicine, Kaohsiung Chang Gung Memorial Hospital and Chang Gung University College of Medicine, Kaohsiung, Taiwan; 2 Division of Cardiology, Department of Internal Medicine, Kaohsiung Chang Gung Memorial Hospital and Chang Gung University College of Medicine, 123, Dapi Road, Niaosung Dist., Kaohsiung City, 83301 Taiwan; 3 Center for Shockwave Medicine and Tissue Engineering, Kaohsiung Chang Gung Memorial Hospital, Kaohsiung, Taiwan; 4 Institute for Translational Research in Biomedicine, Kaohsiung Chang Gung Memorial Hospital, Kaohsiung, Taiwan; 5 Divisions of General Medicine, Department of Internal Medicine, Kaohsiung Chang Gung Memorial Hospital and Chang Gung University College of Medicine, Kaohsiung, Taiwan; 6 Department of Computer Science and Engineering, National Sun Yat-Sen University, Kaohsiung, Taiwan; 7 Department of Healthcare Administration and Medical Informatics, Kaohsiung Medical University, Kaohsiung, Taiwan; 8 Department of Nursing, Asia University, Taichung, Taiwan; 9 Regenerative Medicine and Cell Therapy Research Center, Kaohsiung Medical University, No. 100, Shih-Chuan 1st Road, Kaohsiung, 807 Taiwan; 10 Orthopaedic Research Center, Kaohsiung Medical University, Kaohsiung, Taiwan; 11 Post-Baccalaureate Program in Nursing, Asia University, Taichung, Taiwan; 12 Department of Medical Research, China Medical University Hospital, China Medical University, Taichung, Taiwan; 13 Division of Cardiology, Department of Internal Medicine, Xiamen Chang Gung Hospital, Xiamen, Fujian China

**Keywords:** Sepsis syndrome, Acute kidney injury, Antibiotics, Induced pluripotent stem cell-derived mesenchymal stem-cells, Inflammation, Oxidative stress, Mortality

## Abstract

**Background:**

This study tested whether human induced-pluripotent stem-cell-derived mesenchymal-stem-cells (iPS-MSCs) would offer an additional benefit to the rodent with acute kidney injury (AKI) (ischemia for 1 h followed by reperfusion for 120 h) associated sepsis syndrome (SS) (by cecal-ligation-puncture immediately after AKI-induction) undergoing ciprofloxacin therapy.

**Results:**

Male-adult SD rats (*n* = 80) were categorized into group 1 (sham-operated-control, *n* = 10), group 2 (AKI + SS, *n* = 24), group 3 (AKI + SS + ciprofloxacin/3 mg/kg, orally for 120 h, *n* = 12), group 4 (AKI + SS + iPS-MSCs/1.2 × 10^6^/intravenously administered by 3 h after AKI, *n* = 12), group 5 (AKI + SS + iPS-MSCs/1.2 × 10^6^/intravenously administered by 18 h after AKI, *n* = 12), group 6 (AKI + SS + iPS-MSCs/1.2 × 10^6^/intravenously administered by 3 h after AKI induction + ciprofloxacin, *n* = 10] and euthanized by 120 h. The result showed that the mortality was significantly higher in group 2 than in other groups (all *p* < 0.01). The creatinine level was highest in group 2, lowest in group 1, significantly lower in group 6 than in groups 3, 4 and 5, (all *p* < 0.0001), but it showed no difference among the latter 3 groups. Flow cytometric analysis showed that the circulatory inflammatory cells (Ly6G/CD11^b/c^), early (AN-V+/PI−)/late (AN-V+/PI+) apoptosis, and circulatory/splenic immune cells (CD3+/CD4+, CD3+/CD8a+) were highest in group 2, lowest in group 1, significantly lower in group 6 than in groups 3/4/5 and significantly lower in group 4 than in groups 3/5 (all *p* < 0.0001), but they showed no difference between groups 3/5. Protein expressions of oxidative-stress (NOX-1/NOX2/oxidized protein), apoptotic (cleaved-caspase3/cleaved-PARP/mitochondrial-Bax), fibrotic (TGF-ß/Smad3), inflammatory (MMP-9/IL-6/TNF-α) and autophagic (Atg5/Beclin) biomarkers in kidney exhibited an identical pattern of circulatory inflammatory cells (all *p* < 0.0001).

**Conclusion:**

Combined iPS-MSCs-ciprofloxacin therapy was superior to either one alone for protecting AKI complicated by SS.

**Supplementary Information:**

The online version contains supplementary material available at 10.1186/s13287-021-02582-5.

## Introduction

Acute kidney injury (AKI) commonly takes place in critically ill patients, especially in those of adult intensive care unit (ICU) patients. With the integration of consensus AKI definition criteria from Risk, Injury, Failure, Loss, End-Stage Kidney Disease (RIFLE), Acute Kidney Injury Network (AKIN), and the recent Kidney Disease Improving Global Outcomes (KDIGO), incidence of AKI in adult ICU has been estimated to be around for 16–67% [1–11]. Clinically observational studies have even more shown that in a large 10-year cohort study that enrolled more than 90,000 patients from more than 20 ICUs, AKI incidence increased by 2.8% per year [3], highlighting that this disease entity is in the increasing process worldwide.

The fundamental causes of AKI have been keenly surveyed from many perspectives [4–15], including toxic substances such as chemical compounds and medicines, sepsis syndrome (SS)-associated ischemic kidney injury and acute ischemia–reperfusion (IR) injury, contrast medium, obstruction of the urinary tract, chronic advanced heart failure, hepato-renal syndrome, hypoperfusion/shock, etc. Additionally, acute kidney IR injury has been clearly identified as one of the crucial contributors of AKI [4, 5, 11, 16], and majority of the causal etiologies of AKI can also contribute to acute IR injury in kidney [2, 16–19]. Furthermore, SS is one of the most common contributors for worsening an acute IR injury in kidney, highlighting an extremely strong positive correlation between SS-kidney IR injury and AKI.

It is well known that sepsis/SS is a principal contributor of critical illness [6, 20–23]. Additionally, SS-associated AKI frequency develops at a high incidence rate in critically ill patients [24, 25]. Importantly, the occurrence of AKI in the SS heightens the risk of in-hospital mortality six to eight-fold [25, 26], and the risk of progression to chronic kidney disease is quite common among survivors [27]. Despite this, the mechanistic basis by which sepsis induces AKI are incompletely understood, and hence the update treatment is still reactive and nonspecific.

Plentiful data have demonstrated that mesenchymal stem cell (MSC) therapy efficaciously ameliorated ischemia-related organ dysfunction mainly through its anti-inflammatory, immunomodulatory and tissue regenerative properties [2, 28–30]. Additionally, our previous series of preclinical studies have shown that MSC therapy also is an attractive and promising modality for treatment of SS in rodents [31–33]. On the other hand, the use of human induced pluripotent stem cell derived mesenchymal stem cells (iPS-MSCs) has recently appeared as an innovative modality for regenerative medicine [34, 35] and also acts as a therapeutic possibility for different disease entities [35–37] in numerous pre-clinical studies. The great potentiality for future clinical application of iPSC-MSCs has been extensively discussed by scientists due to this type of cells is easily accessible by standardized nuclear transfer technique. Thus, the inexhaustible iPSC-MSCs can be easily obtained through differentiation, re-expansion and maintenance. Additionally, it is well recognized that the capacity of anti-inflammation and immunomodulation of human iPSC-MSCs is not inferior to other sources of MSCs. Based on the aforementioned issues, we proposed that combined iPSC-MSCs and ciprofloxacin (i.e., an antibiotic) might be superior to either single therapy for treating the SS-associated AKI in rodent.

## Materials and methods

### Ethics

All animal procedures were approved by the Institute of Animal Care and Use Committee at Kaohsiung Chang Gung Memorial Hospital (Affidavit of Approval of Animal Use Protocol No. 2019090901) and performed in accordance with the Guide for the Care and Use of Laboratory Animals.

Animals were housed in an Association for Assessment and Accreditation of Laboratory Animal Care International (AAALAC; Frederick, MD, USA)-approved animal facility in our hospital with controlled temperature and light cycles (24 °C and 12/12 light cycle).

### The procedures of cecal ligation and puncture (CLP) for induction of sepsis syndrome (SS)

Rats were anesthetized with inhalational 2.0% isoflurane and placed in a supine position on a warming pad at 37 °C with the abdomen shaved. Under sterile conditions, the abdominal skin and muscle were incised, and the cecum was exposed in all animals. In the sham-operated control (SC), the abdomen was then closed, and the animals were allowed to recover from anesthesia. In the experimental CLP groups, the cecum of each animal was prolene suture ligated over its distal portion (i.e., distal ligation) and the cecum distal to the ligature was punctured twice with an 18G needle and squeezed for allowing the cecal contents released into peritoneum, as we previously described [31, 33]. The abdominal muscle and skin were sutured, and the animals were then allowed to recover from anesthesia.

### Acute kidney ischemia–reperfusion (AKIR) procedure

The procedure and protocol of AKIR procedure have been described in our previous report [35]. Briefly, animals were anesthetized by inhalational 2.0% isoflurane, placed supine on a warming pad at 37 °C for midline laparotomy. The SC received laparotomy only, while AKIR of both kidneys was induced in all animals by clamping the renal pedicles for one hour using non-traumatic vascular clips. The animals in each group were euthanized and the kidneys were harvested for individual study by day 5 after the IR procedure. The procedure of AKIR was immediately performed after SS induction.

### Rationale of AKIR induction in SS rat for the purpose of this preclinical study

In our daily clinical practice, it is well recognized SS is frequently observed in patients who are admitted for AKI, especially in those of the elder, hematological disorder, cancer and immune-compromised 8607 patients (i.e., this was defined as AKI developed, then followed by SS rather the phenomenon of SS induced AKI). This was the reason for we created an animal model of AKI procedure + SS induction rather only SS-induced AKI.

### Animal grouping

Pathogen-free, adult male Sprague–Dawley (SD) rats (*n* = 80) weighing 325–350 g (Charles River Technology, BioLASCO Taiwan Co. Ltd., Taiwan) were divided into 6 groups: group 1 [sham-operated control (SC) received laparotomy only, plus intra-peritoneal administration of 2.0 mL normal saline at 3 h after IR procedure, *n* = 10)], group 2 (SS + AKIR, *n* = 24), group 3 [SS + AKIR + ciprofloxacin (3.0 mg/kg, orally, initial dosage at 3 h after SS-AKIR induction, *n* = 12) for 5 days], group 4 [SS + AKIR + iPS-MSCs (1.2 × 10^6^ cells/rat by intravenous injection at 3 h, i.e., defined as early treatment, *n* = 12) after SS-AKIR induction], group 5 [SS + AKIR + iPS-MSCs (1.2 × 10^6^ cells/rat by intravenous injection at 18 h, i.e., defined as late treatment, *n* = 12) after SS-AKIR induction] and group 6 [SS + AKIR + ciprofloxacin + iPS-MSCs (at 3 h after SS-AKIR induction, *n* = 10)]. The dosage of ciprofloxacin was based on our previous report [40]. On the other hand, the culturing and dosage of iPS-MSCs were based on our recent report [36].

### Methodology of in vitro study of cell culturing for differentiation of human iPSC into mesenchymal stem cells (MSCs)

The procedure and protocol of human iPSC culture for differentiation into MSCs were based on our previous study [35] and detailed in formation was illustrated as Additional file 1: Fig. 1. In details, at day 1, the human iPSCs [mTeSR™1; StemCell, #28315) were first washed by 5 mL PBS, followed by 2 mL Accutase (Gibco, #A1110501; Accutase: PBS = 1:1); the incubator reaction continued for 1 min. The 2 mL KO DMEM/F12 (Gibco, #12660012) was added and the cells were collected in 15 mL centrifuge tubes for 5-min duration of centrifuge (× 200*g*). The cells were then cultured in a 10-cm dish for 24 h in mTeSR™1 culture medium.

By day 2, the cells (mTeSR™1) were collected and washed by 5 mL PBS. STEMdiff™-ACF Mesenchymal Induction Medium (StemCell, #05241) was added into incubator for culture and proceeded for 24 h. The STEMdiff™-ACF Mesenchymal Induction Medium was renewed once per day from days 1 to 3. This procedure was repeated on days 3 to 6. On days 7 to 21, the procedure was repeated but the culture medium was refreshed every 3 days.

To prove the cells were workably and could differentiate into adipocytes, chondrocytes, osteoblast, we finally repeated the culture with different culture medium and the results were illustrated in Additional file 2: Fig. 2 and Additional file 3: Fig. 3.

### Assessment of serum creatinine and BUN Levels, and collection of 24h urine for the ratio of urine protein to urine creatinine at 72 h after SS-AKIR procedure

Blood samples were collected from all animals in each group for assessing changes in serum creatinine and blood urine nitrogen (BUN) levels at days 0 prior to SS-AKIR procedure and at days 1 and 5 after SS-AKIR procedure.

For the collection of 24h urine for individual study, each animal was put into a metabolic cage [DXL-D, space: 190 × 290 × 550, Suzhou Fengshi Laboratory Animal Equipment Co. Ltd., Mainland China] for 24 h with free access to food and water. Urine in 24 h was collected from all animals at day 0 and from initial day 4 to final day 5 (i.e., total 24 h) after the AKIR procedure for determining the ratio of urine protein to urine creatinine (Ra-Up/Uc).

### End of study period

The animals in each group were sacrificed at day 5 after SS-AKIR induction. The blood samples from circulation and spleen were collected for flow cytometric analyses to determine the levels of inflammatory cells, apoptotic mononuclear and immune cells. The kidneys in each group of animals were harvested for individual study.

### Qualitative analysis of kidney injury scores at day 5 after SS-AKIR procedure

Histopathology scoring of kidney injury was assessed in a blinded fashion as we previously reported [35, 37]. Briefly, kidney specimens from all animals were fixed in 10% buffered formalin, embedded in paraffin, sectioned at 5 μm and stained with hematoxylin and eosin (H&E) for light microscopy. The scoring system reflected the grading of tubular necrosis, loss of brush border, cast formation, and tubular dilatation in 10 randomly chosen, non-overlapping fields (200x) as follows: 0 (none), 1 (≤ 10%), 2 (11–25%), 3 (26–45%), 4 (46–75%), and 5 (≥ 76%).

### Flow cytometric by day 5 after SS-AKIR procedure

Circulating and splenic levels of CD3+/CD4+ cells (BD Biosciences), CD3+/CD8a+ cells (BD Biosciences) and Treg + cells (BD Biosciences), three indicators of immune cells, and CD11a/b + cells (BD Biosciences) and Ly6G + cells (BD Biosciences), two indices of inflammatory cells, as well as early (AN-V+/PI−) and late (AN-V+/PI+) (BD Biosciences) mononuclear apoptotic cells were studied by flow cytometric analysis.

### Immunohistochemical (IHC) and Immunofluorescent (IF) Staining

The procedure and protocol for IHC and IF staining have been described in our previous reports [36–38]. For IHC and IF staining, rehydrated paraffin sections were first treated with 3% H2O2 and incubated with Immuno-Block reagent (BioSB, Santa Barbara, CA, USA) for 30 min at room temperature. Sections were then incubated with primary antibodies specifically against zonula occludens-1 (ZO-1) (1:200, Abcam), kidney injury molecule (KIM)-1 (1:400, Novus), p-cadherin (1:100, Novus), synaptopodin (1:500, Santa Cruz) and γ-H2AX (1:1000, Abcam), while sections incubated with the use of irrelevant antibodies served as controls. Three sections of kidney specimen from each rat were analyzed. For quantification, three random chosen HPFs (200 × or 400 × for IHC and IF studies) were analyzed in each section. The mean number of positively stained cells per HPF for each animal was then determined by summation of all numbers divided by 9.

An IHC-based or IF-based scoring system was adopted for semi-quantitative analyses of ZO-1, p-cadherin and KIM-1 in the kidney as a percentage of positive cells in a blinded fashion (score of positively-stained cell for these biomarkers as: 0 = negative staining; 1 =  < 15%; 2 = 15–25%; 3 = 25–50%; 4 = 50–75%; 5 =  > 75%-100%/per HPF).

### Western blot analysis

The procedure and protocol for Western blot analysis have been described in our previous reports [35–37]. Briefly, equal amounts (50 μg) of protein extracts were loaded and separated by SDS-PAGE using acrylamide gradients. After electrophoresis, the separated proteins were transferred electrophoretically to a polyvinylidene difluoride (PVDF) membrane (GE, UK). Nonspecific sites were blocked by incubation of the membrane in blocking buffer [5% nonfat dry milk in T-TBS (TBS containing 0.05% Tween 20)] overnight. The membranes were incubated with the indicated primary antibodies [tumor necrosis factor (TNF)-α (1: 1000, Cell Signaling), interleukin (IL)-6 (1:1000, Abcam), matrix metalloproteinase (MMP)-9 (1:1000, Abcam), NOX-1 (1: 1500, Sigma), NOX-2 (1: 1000, Sigma), beclin1 (1:1000, Abcam), Atg5 (1:1000, Abcam), mitochondrial Bax (1:1000, Abcam), cleaved caspase 3 (1: 1000, Cell Signaling), cleaved Poly (ADP-ribose) polymerase (PARP) (1: 1000, Cell Signaling), transforming growth factor (TGF)-ß (1:1000, Abcam) and Smad3 (1: 1000, Cell Signaling) for 1 h at room temperature]. Horseradish peroxidase-conjugated anti-rabbit immunoglobulin IgG (1:2000, Cell Signaling, Danvers, MA, USA) was used as a secondary antibody for one-hour incubation at room temperature. The washing procedure was repeated eight times within one hour. Immunoreactive bands were visualized by enhanced chemiluminescence (ECL; Amersham Biosciences, Amersham, UK) and exposed to Biomax L film (Kodak, Rochester, NY, USA). For quantification, ECL signals were digitized using Labwork software (UVP, Waltham, MA, USA).

### Assessment of oxidative stress

The procedure for assessing the protein expression of oxidative stress was based on our previous reports [37–40]. The Oxyblot Oxidized Protein Detection Kit was purchased from Chemicon (S7150). DNPH derivatization was carried out on 6 μg of protein for 15 min according to the manufacturer’s instructions. One-dimensional electrophoresis was carried out on 12% SDS/polyacrylamide gel after DNPH derivatization. Proteins were transferred to nitrocellulose membranes which were then incubated in the primary antibody solution (anti-DNP 1: 150) for 2 h, followed by incubation in the secondary antibody solution (1:300) for 1 h at room temperature. The washing procedure was repeated eight times within 40 min. Immunoreactive bands were visualized by enhanced chemiluminescence (ECL; Amersham Biosciences) which were then exposed to Biomax L film (Kodak). For quantification, ECL signals were digitized using Labwork software (UVP). For oxyblot protein analysis, a standard control was loaded on each gel.

### Statistical analysis

Quantitative data were expressed as mean ± SD. Statistical analysis was adequately performed by ANOVA followed by Bonferroni multiple-comparison post hoc test. Statistical analysis was performed using SPSS statistical software for Windows version 22 (SPSS for Windows, version 22; SPSS, IL, USA). A value of *p* < 0.05 was considered as statistically significant.

## Results

### iPSC-MSCs possessed the capacities of anti-inflammation and immunomodulation (Fig. 1)

To test whether the iPS-MSCs had properties of downregulation of inflammation and immunogenicity, the cell culture in Transwell was categorized into Group A [raw 264.7 cell line (i.e., murine macrophage cells)], Group B [Raw 264.7 + lipopolysaccharide (LPS) (5 × 10^5^/well co-cultured for 6 h)], Group C [raw 264.7 + iPS-MSC (5 × 10^5^/well)] and Group D (raw 264.7 + LPS + iPS-MSC). The Raw264.7 purchased from BCRC (Bioresource Collection and Research Center, Taiwan)] were put into the bottom compartment, while iPS-MSCs were put into the upper compartment of Transwell for coculture.

The flow cytometric result and the IF staining demonstrated that the M2 to M1 ratio, an indicator of immunomodulation, was highest in Group A, lowest in Group B and significantly higher in group C than in group D, whereas the number of macrophage migration inhibitory factor (MIF) + cells exhibited an opposite pattern of the M2 to M1 ratio among the groups. Furthermore, the protein expressions of TNF-α and IL-6, two indicators of proinflammatory cytokine, showed a similar pattern, whereas the protein expression of IL-10, an indicator of anti-inflammation, exhibited an opposite pattern of MIF among the four groups. Our in vitro results proved that iPS-MSCs had strong capacities of anti-inflammation and immunomodulation. Based on these findings, we then performed an animal model study.

**Fig. 1:**
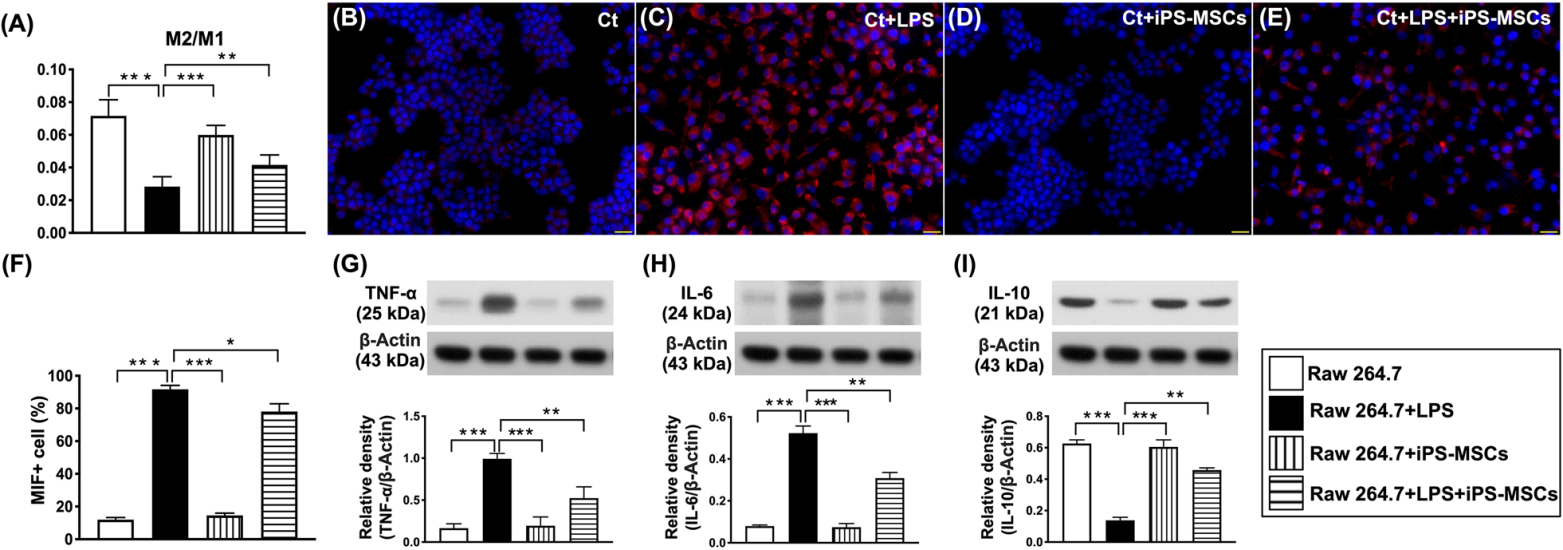
Impact of iPSC-MSCs treatment on enhancing the capacity of anti-inflammation and immunomodulation. **A** Flow-cytometric analytical result of M2 to M1 ratio. **B–E** Immunofluorescent (IF) microscopic finding (400x) for identification of positively-stained macrophage migration inhibitory factor (MIF) cells (green color). Scale bars in right lower corner represent 20 µm. **F** Analytical result of number of MIF + cells. **G** Protein expression of tumor necrosis factor (TNF-α). **H** Protein expression of interleukin (IL)-6. **I** Protein expression of IL-10. *n* = 4 or 6 for each group. * indicates *p* value < 0.05; ** indicates *p* value  < 0.01; *** indicates *p* value  < 0.001. iPS-MSCs = induced pluripotent stem cells-derived mesenchymal stem cells; LPS = lipopolysaccharide

### Circulating levels of inflammatory cells, immune cells and apoptotic mononuclear cells, and mortality rate by day 5 after SS-AKIR procedure (Fig. 2)

To elucidate whether the impact of early and late iPS-MSCs treatments would affect the immune and inflammatory reaction and the prognostic outcome, the animals were categorized into six groups: i.e., group 1 (SC), group 2 (SS + AKIR), group 3 (SS + AKIR + ciprofloxacin), group 4 (SS + AKIR + iPS-MSC/early treatment at 3 h), group 5 (SS + AKIR + iPS-MSC/late treatment at 18 h) and group 6 (SS + AKIR + ciprofloxacin + iPS-MSC).

**Fig. 2:**
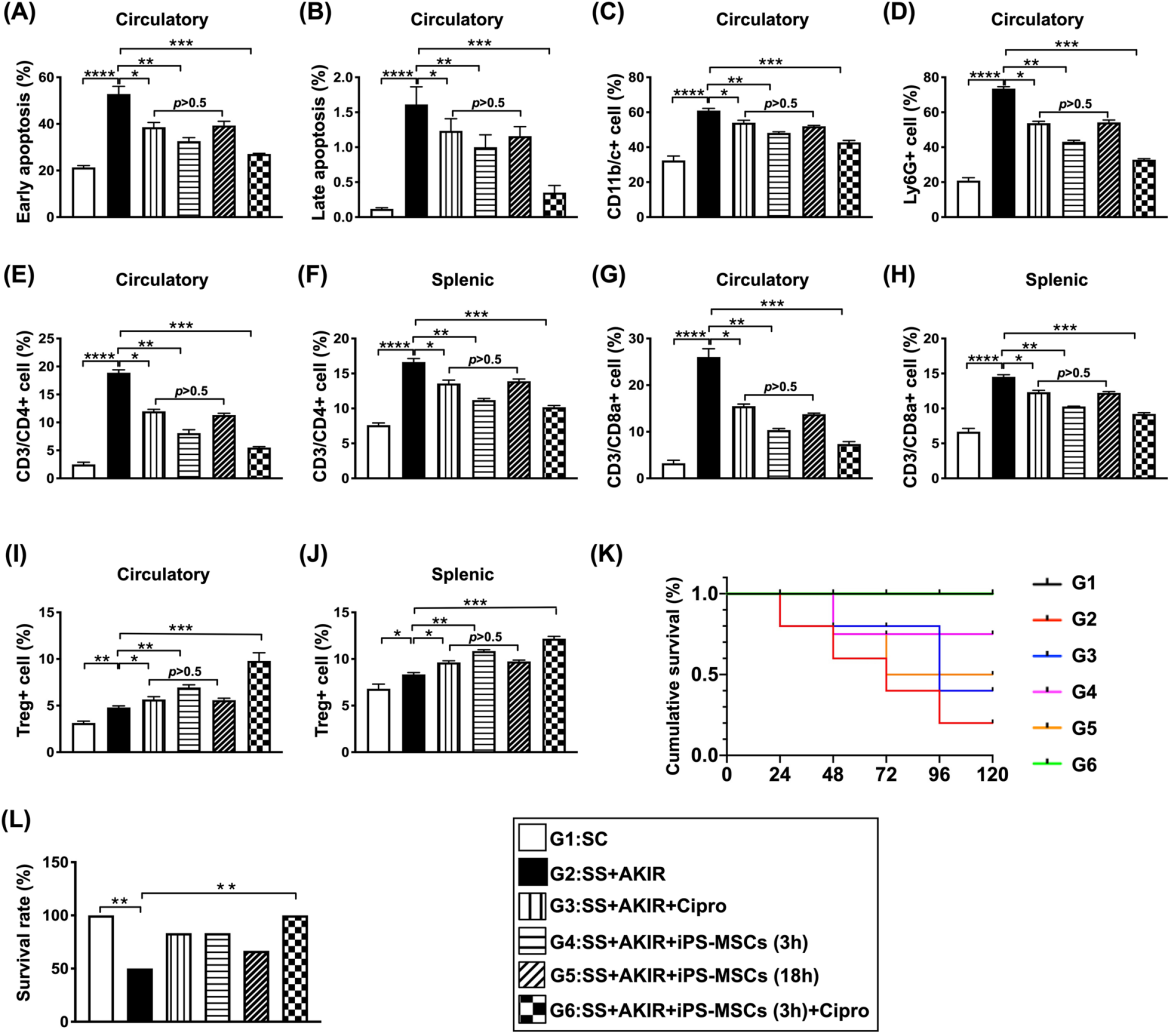
Flow cytometric analyses for determining the circulating levels of inflammatory cells, immune cells and apoptotic mononuclear cells, and mortality rate by day 5 after SS-AKIR procedure. **A** Analytical result of number of early apoptosis (AN-V+/PI−) of circulatory mononuclear cells. **B** Analytical result of number of late apoptosis (AN-V+/PI+) of circulatory mononuclear cells. **C** Analytical result of circulatory number of CD11b/c + cells. **D** Analytical result of circulatory number of Ly6G + cells. **E** Analytical result of circulatory number of CD3/CD4+ cells. **F** Analytical result of splenic number of CD3/CD4+ cells. **G** Analytical result of circulatory number of CD3/CD8a+ cells. **H** Analytical result of splenic number of CD3/CD8a+ cells. **I** Analytical result of circulatory number of Treg+ (i.e., CD4+/CD25+ /Foxp3 +) cells. **J** Analytical result of splenic number of Treg + cells. **K** Illustrating the K-M survival curve. **L** The mortality rate. *n* = 6 for each group; on the other hand, for mortality analysis: *n* = 10 in G1; *n* = 24 in G2; *n* = 12 in G3; *n* = 12 in G4; *n* = 12 in G5; *n* = 10 in G6. * indicates *p* value  < 0.05; ** indicates *p* value  < 0.01; *** indicates *p* value  < 0.001; **** indicates *p* value  < 0.0001. G2 = sepsis syndrome (SS) + acute kidney ischemia–reperfusion (AKIR); G3 (SS + AKIR + ciprofloxacin administered at 3 h after SS-AKIR induction); G4 [SS + AKIR + iPS-MSCs by intravenous injection at 3 h, after SS-AKIR (i.e., defined as early treatment)]; G5 [SS + AKIR + iPS-MSCs by intravenous injection at 18 h after SS-AKIR (i.e., defined as late treatment) after SS-AKIR induction]; G6 [SS + AKIR + ciprofloxacin and iPS-MSCs at 3 h after SS-AKIR induction]. iPS-MSCs = induced pluripotent stem cells-derived mesenchymal stem cells

As we expected, the flow cytometric analysis showed that the numbers of early and late apoptotic mononuclear cells in circulation were lowest in group 1, highest in group 2, significantly lower in group 6 than in groups 3 to 5 and significantly lower in group 4 than in groups 3 and 5, but they did not differ between groups 3 and 5. Additionally, the circulating and splenic levels of CD3/CD4+ cells and CD3/CD8a+ cells, two indicators of immune cells, exhibited an identical pattern of apoptotic cells among the six groups. On the other hand, the circulating and splenic levels of Treg+ (i.e., CD4+/CD25+/Foxp3+ cells, an index of immune regulating cells, exhibited a progressively increasing pattern from groups 1 to 6.

The mortality rate by day 5 was significantly higher in group 2 than in groups 1 and 6. However, this parameter did not differ among the groups 1 and 3 to 6 or groups 2 to 5.

### Time courses of circulating levels of creatinine and blood urine nitrogen (BUN) and ratio of urine protein to urine creatinine (Ra-Up/Uc) (Fig. 3)

The baseline levels of BUN, creatinine and Ra-Up/Uc did not differ among the six groups. However, by days 1 and 5 after SS-AKIR procedure, the BUN and creatinine and by day 5 the Ra-Up/Uc, were lowest in group 1, highest in group 2, significantly lower in group 6 than in groups 3 to 5 and significantly lower in group 4 than in groups 3 and 5, but they did not differ between groups 3 and 5.

**Fig. 3:**
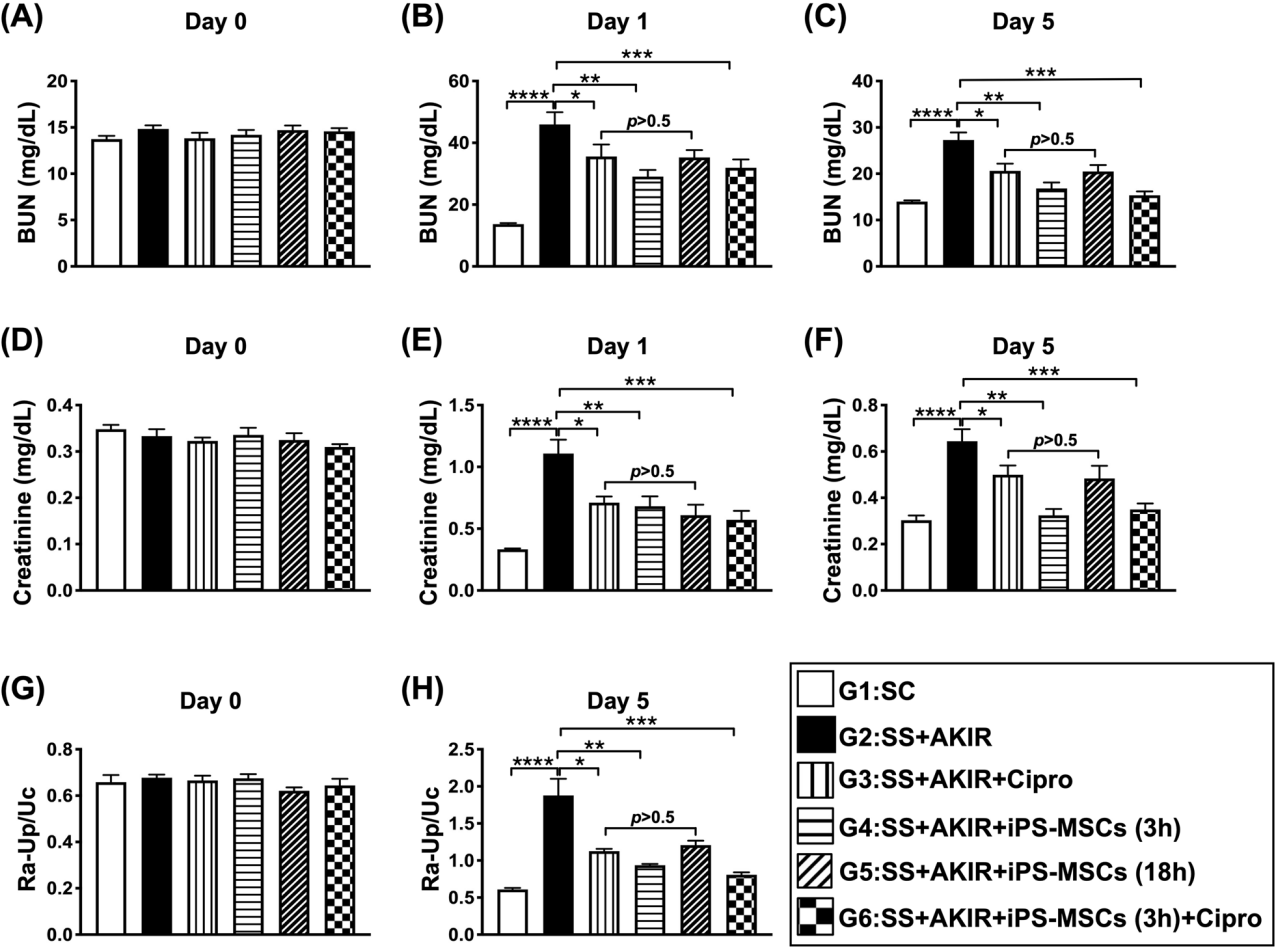
Time courses of circulating levels of creatinine and blood urine nitrogen (BUN) and ratio of urine protein to urine creatinine (Ra-Up/Uc). **A** Circulating level of BUN at day 0, *p* > 0.5. **B** Circulating level of BUN at day 1. **C** Circulating level of BUN at day 5. **D** Circulating level of creatinine by day 0, *p* > 0.5. **E** Circulating level of creatinine at day 1. **F** Circulating level of creatinine at day 5. **G** Ra-Up/Uc by day 0, *p* > 0.5. **H** Ra-Up/Uc by day 5. *n* = 8 or 12 for each group. * indicates *p* value  < 0.05; ** indicates *p* value  < 0.01; *** indicates *p* value  < 0.001; **** indicates *p* value  < 0.0001. G1 = (sham-operated control); G2 = sepsis syndrome (SS) + acute kidney ischemia–reperfusion (AKIR); G3 (SS + AKIR + ciprofloxacin administered at 3 h after SS-AKIR induction); G4 [SS + AKIR + iPS-MSCs by intravenous injection at 3 h, after SS-AKIR (i.e., defined as early treatment)]; G5 [SS + AKIR + iPS-MSCs by intravenous injection at 18 h after SS-AKIR (i.e., defined as late treatment) after SS-AKIR induction]; G6 [SS + AKIR + ciprofloxacin and iPS-MSCs at 3 h after SS-AKIR induction]. iPS-MSCs = induced pluripotent stem cells-derived mesenchymal stem cells

The accumulative mortality rate by day 5 was significantly higher in groups 2 and 3 than groups 1, 4 and 5, but it showed no difference between groups 2 and 3 or among groups 1, 4 and 5.

### Kidney injury score and DNA-damaged marker by day 5 after SS-AKIR procedure (Fig. 4)

**Fig. 4:**
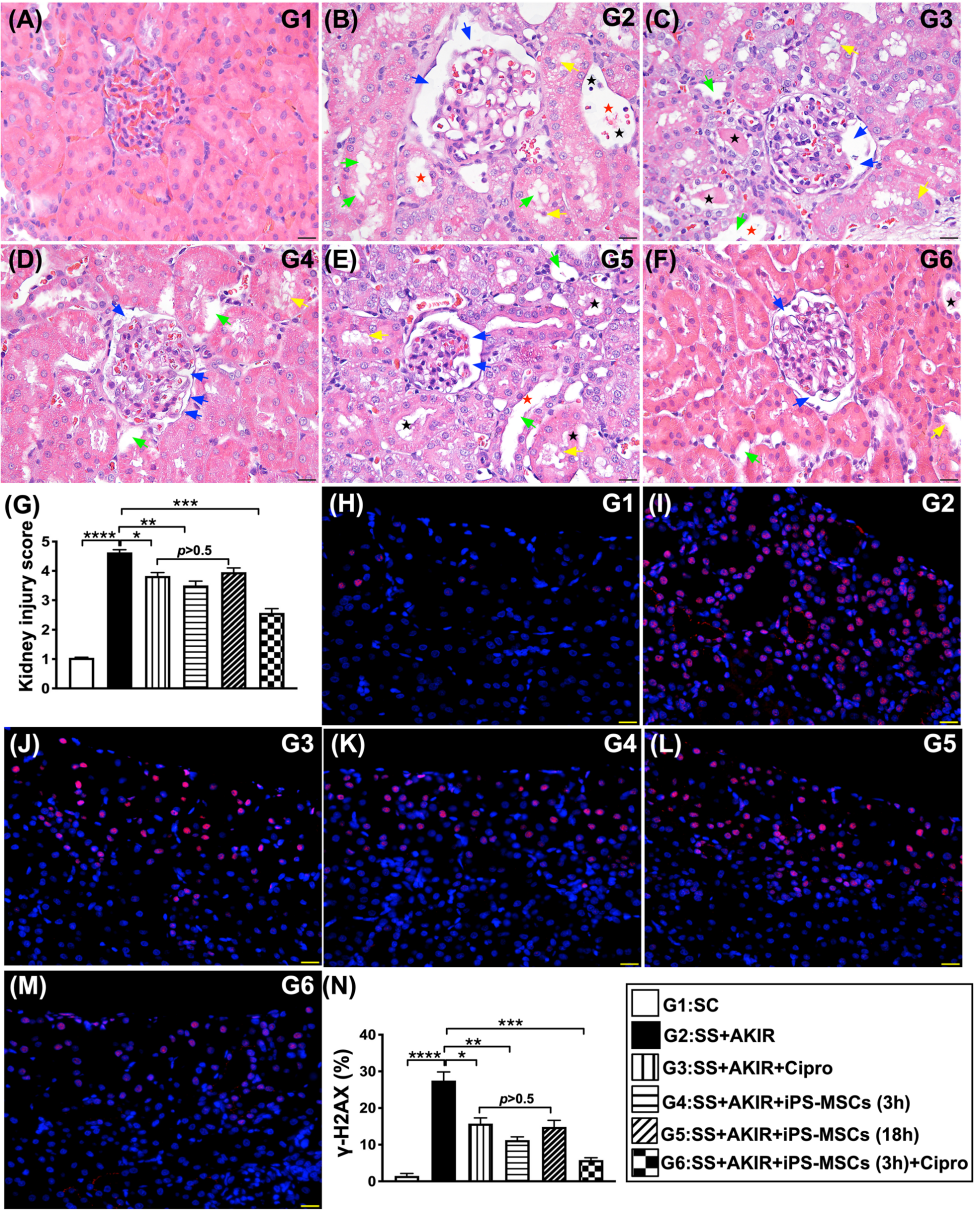
Kidney injury score and DNA-damaged marker by day 5 after SS-AKIR procedure. A–F Light microscopic findings (200x; H&E stain) showing significantly higher loss of brush border in renal tubules (yellow arrows), tubular necrosis (green arrows), tubular dilatation (red asterisk), protein cast formation (black asterisk), and dilatation of Bowman’s capsule (blue arrows) in IR group than in other groups. Scale bars in right lower corner represent 50 µm. H–M Illustrating the immunofluorescent (IF) microscopic finding (400x) for identification of γ-H2AX + cells (red color). N Analytical result of number of γ-H2AX + cells. Scale bars in right lower corner represent 20 µm. *n* = 6 for each group. * indicates *p* value  < 0.05; ** indicates *p* value  < 0.01; *** indicates *p* value  < 0.001; **** indicates *p* value  < 0.0001. G1 = (sham-operated control); G2 = sepsis syndrome (SS) + acute kidney ischemia–reperfusion (AKIR); G3 (SS + AKIR + ciprofloxacin administered at 3 h after SS-AKIR induction); G4 [SS + AKIR + iPS-MSCs by intravenous injection at 3 h, after SS-AKIR (i.e., defined as early treatment)]; G5 [SS + AKIR + iPS-MSCs by intravenous injection at 18 h after SS-AKIR (i.e., defined as late treatment) after SS-AKIR induction]; G6 [SS + AKIR + ciprofloxacin and iPS-MSCs at 3 h after SS-AKIR induction]. iPS-MSCs = induced pluripotent stem cells-derived mesenchymal stem cells

The kidney injury score was lowest in group 1, highest in group 2, significantly lower in group 6 than in groups 3 to 5 and significantly lower in group 4 than in groups 3 and 5, but it did not differ between groups and 3 and 5. Additionally, the cellular expression of γ-H2AX, an indicator of DNA-damaged biomarker, exhibited an identical pattern of kidney injury score among the six groups.

### Glomerular ultrastructural expressions by day 5 after SS-AKIR procedure (Fig. 5)

**Fig. 5:**
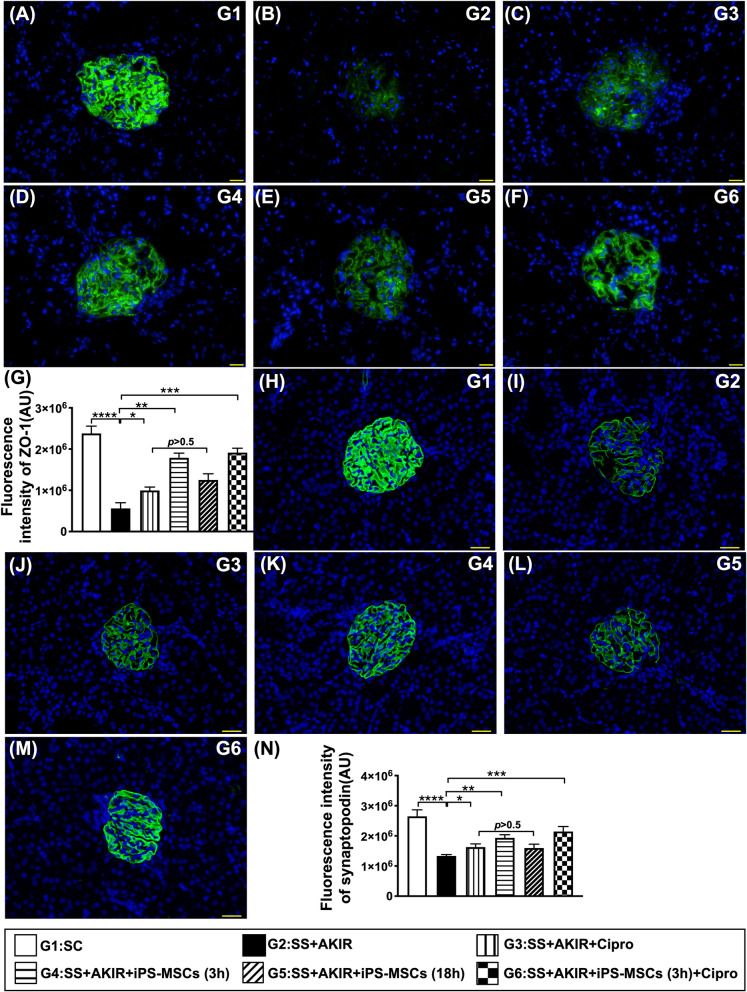
Glomerular ultrastructural expressions by day 5 after SS-AKIR procedure. A–F Illustrating immunofluorescent (IF) microscopic finding (400x) for identification of positively-stained zonula occludens-1 (ZO-1) in renal glomerulus (green color). G) Analytical result of ZO-1 expression. H–M Illustrating IF microscopic finding (400x) for identification of synaptopodin in renal glomerulus (green color). N Analytical results of synaptopodin expression. *n* = 6 for each group). * indicates *p* value  < 0.05; ** indicates *p* value  < 0.01; *** indicates *p* value  < 0.001; **** indicates *p* value  < 0.0001. G1 = (sham-operated control); G2 = sepsis syndrome (SS) + acute kidney ischemia–reperfusion (AKIR); G3 (SS + AKIR + ciprofloxacin administered at 3 h after SS-AKIR induction); G4 [SS + AKIR + iPS-MSCs by intravenous injection at 3 h, after SS-AKIR (i.e., defined as early treatment)]; G5 [SS + AKIR + iPS-MSCs by intravenous injection at 18 h after SS-AKIR (i.e., defined as late treatment) after SS-AKIR induction]; G6 [SS + AKIR + ciprofloxacin and iPS-MSCs at 3 h after SS-AKIR induction]. iPS-MSCs = induced pluripotent stem cells-derived mesenchymal stem cells

IF microscopic finding demonstrated that cellular expression of ZO-1, a tight junction-associated protein which provides a link between the integral membrane proteins and the filamentous cytoskeleton in podocytes, was highest in group 1, lowest in group 2, significantly higher in group 6 than in groups 3 to 5, and significantly higher in group 4 than in groups 3 and 5, but it was similar between groups 3 and 5. Additionally, the expression of synaptopodin, predominantly in glomerulus, a component of podocyte foot process, displayed an identical patten of ZO-1 among the six groups.

### Kidney injury biomarker and podocyte component expressed in glomerulus by day 5 after SS-AKIR procedure (Fig. 6)

**Fig. 6:**
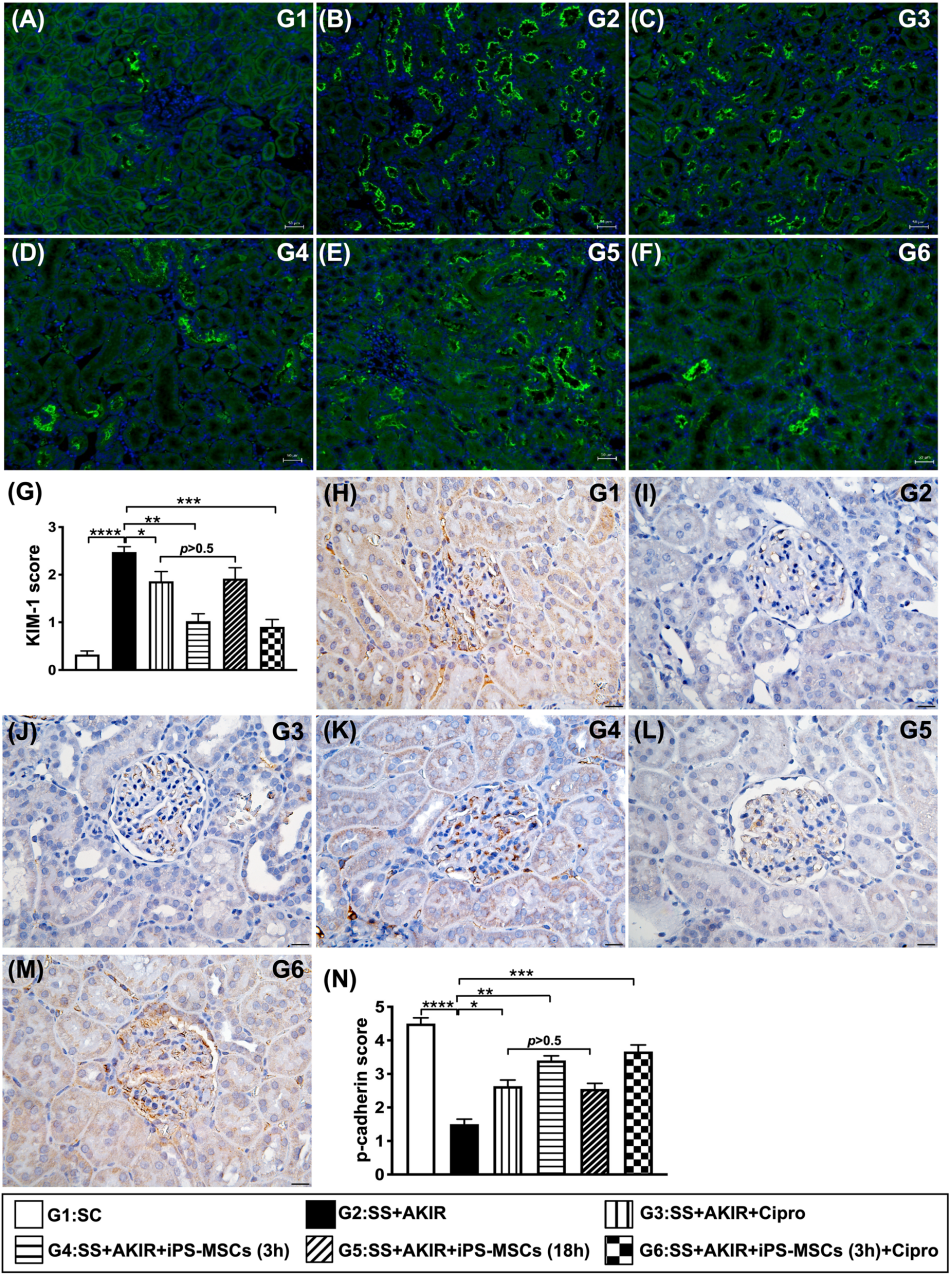
Kidney injury biomarker and podocyte component expressed in glomerulus by day 5 after SS-AKIR procedure. A–F Showing the immunofluorescent microscopic finding (200x) for identification of kidney injury molecule (KIM)-1 in renal tubules (green color). G Analytical results of KIM-1 expression. Scale bars in right lower corner represent 50 µm. H–M Illustrating the microscopic finding (200x) of immunohistochemical staining for identification of p-cadherin in glomeruli (gray color). N Analytical result of p-cadherin expression. Scale bars in right lower corner represent 20 µm. *n* = 6 for each group). * indicates *p* value  < 0.05; ** indicates *p* value  < 0.01; *** indicates *p* value  < 0.001; **** indicates *p* value  < 0.0001. G1 = (sham-operated control); G2 = sepsis syndrome (SS) + acute kidney ischemia–reperfusion (AKIR); G3 (SS + AKIR + ciprofloxacin administered at 3 h after SS-AKIR induction); G4 [SS + AKIR + iPS-MSCs by intravenous injection at 3 h, after SS-AKIR (i.e., defined as early treatment)]; G5 [SS + AKIR + iPS-MSCs by intravenous injection at 18 h after SS-AKIR (i.e., defined as late treatment) after SS-AKIR induction]; G6 [SS + AKIR + ciprofloxacin and iPS-MSCs at 3 h after SS-AKIR induction]. iPS-MSCs = induced pluripotent stem cells-derived mesenchymal stem cells

The IF microscopic finding demonstrated that the cellular expression of KIM-1, a kidney injury biomarker predominantly expressed in renal tubules, was lowest in group 1, highest in group 2, significantly lower in group 6 than in groups 3 to 5, and significantly lower in group 4 than in groups 3 and 5, but it did not differ between groups 3 and 5. On the other hand, the change in the cellular expression P-cadherin, predominantly in renal glomerulus and colocalized with ZO-1, exhibited an opposite pattern of KIM-1 among the six groups.

### Protein expressions of oxidative stress, autophagic biomarkers, apoptosis and fibrosis in kidney by day 5 after SS-AKIR procedure (Figs. 7 and 8)

**Fig. 7:**
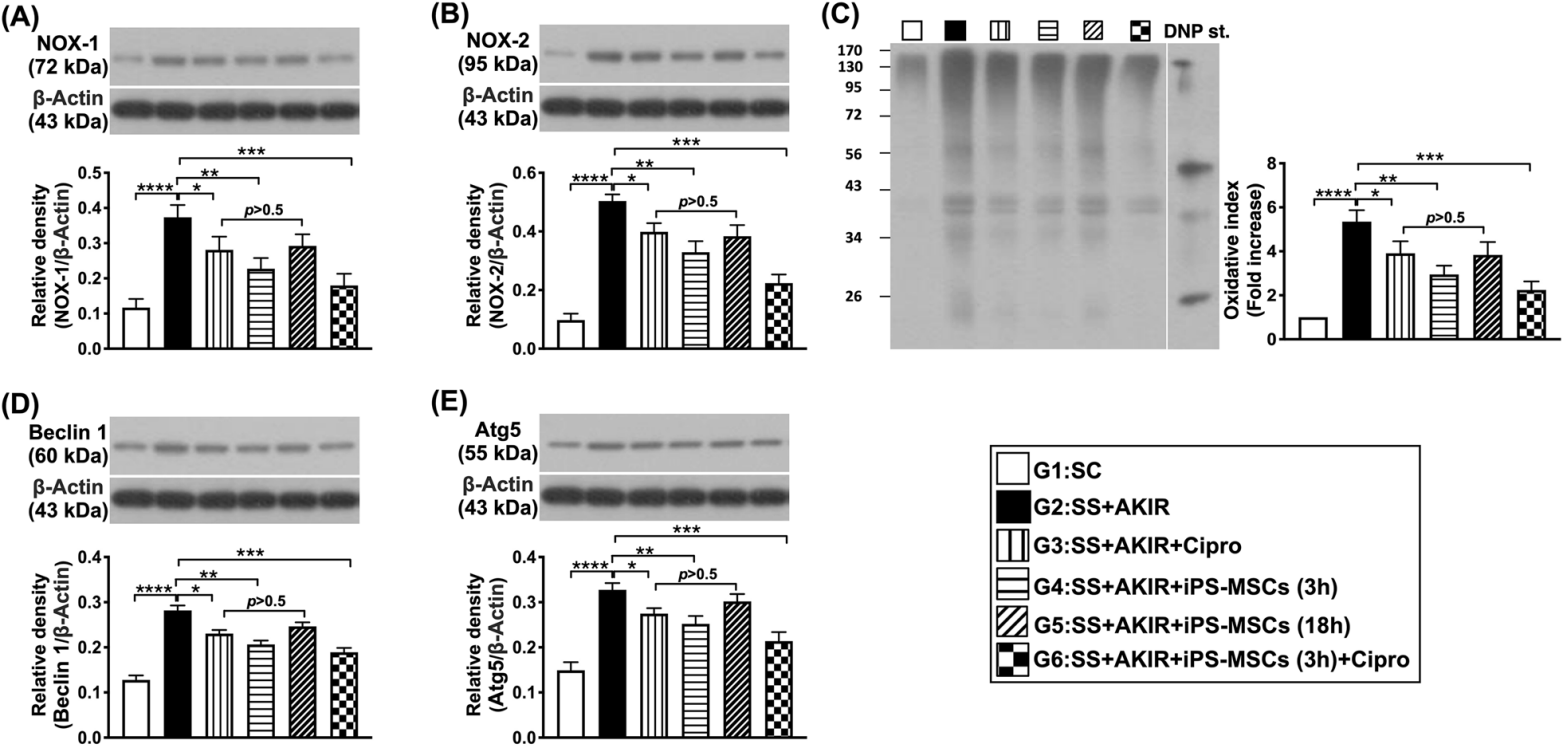
Protein expressions of oxidative stress and autophagic biomarkers by day 5 after SS-AKIR procedure. A Protein expression of NOX-1. B Protein expression of NOX-2. C The oxidized protein expression. (Note: the right and left lanes shown on the upper panel represent protein molecular weight marker and control oxidized molecular protein standard, respectively). M.W. = molecular weight; DNP = 1–3 dinitrophenylhydrazone. D Protein expression of beclin 1. E Protein expression of Atg5. *n* = 6 for each group. * indicates *p* value  < 0.05; ** indicates *p* value  < 0.01; *** indicates *p* value  < 0.001; **** indicates *p* value  < 0.0001. G1 = (sham-operated control); G2 = sepsis syndrome (SS) + acute kidney ischemia–reperfusion (AKIR); G3 (SS + AKIR + ciprofloxacin administered at 3 h after SS-AKIR induction); G4 [SS + AKIR + iPS-MSCs by intravenous injection at 3 h, after SS-AKIR (i.e., defined as early treatment)]; G5 [SS + AKIR + iPS-MSCs by intravenous injection at 18 h after SS-AKIR (i.e., defined as late treatment) after SS-AKIR induction]; G6 [SS + AKIR + ciprofloxacin and iPS-MSCs at 3 h after SS-AKIR induction]. iPS-MSCs = induced pluripotent stem cells-derived mesenchymal stem cells

**Fig. 8:**
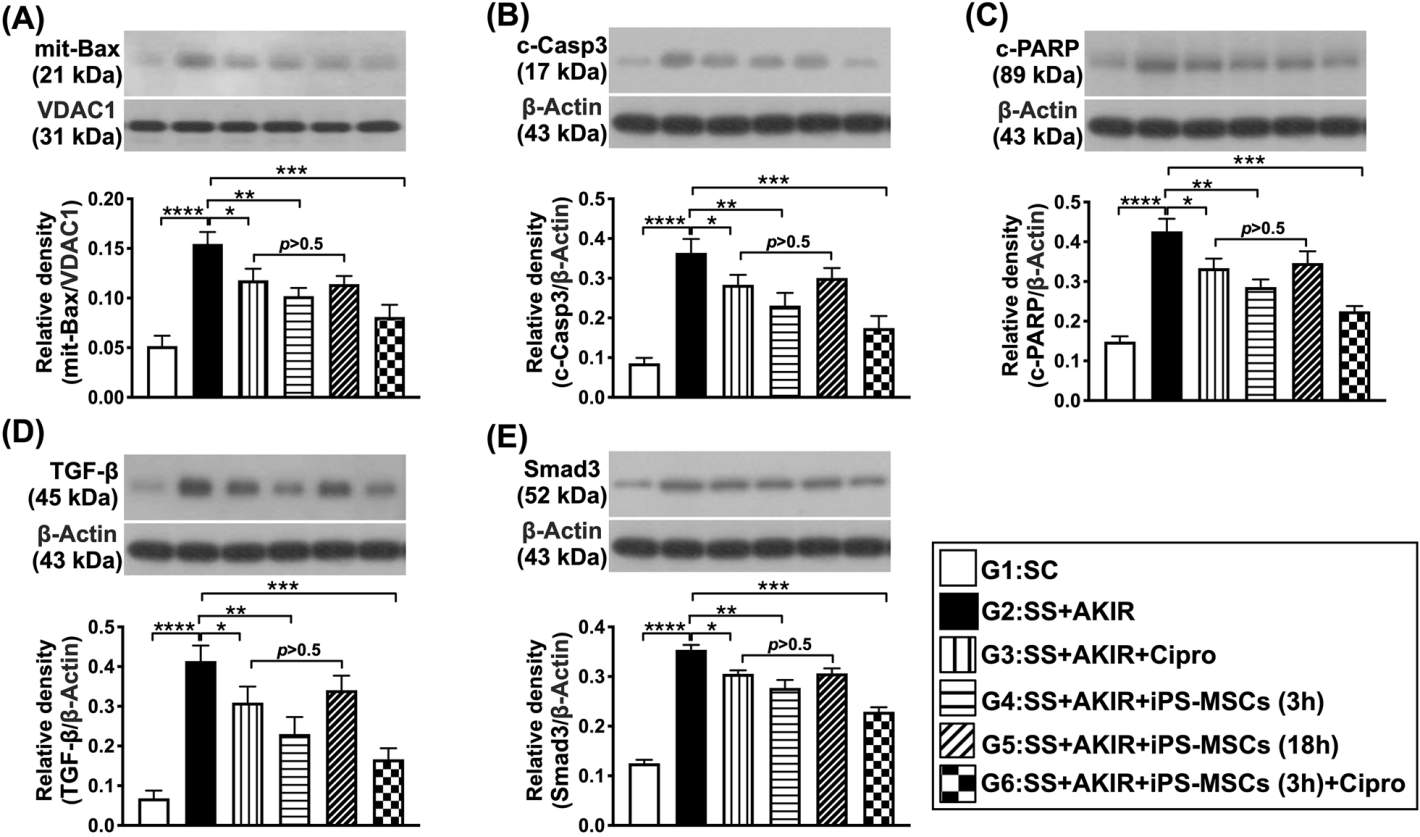
Protein expressions of apoptosis and fibrosis in kidney by day 5 after SS-AKIR procedure. A Protein expression of mitochondrial (mit)-Bax. B Protein expression of cleaved caspase 3 (c-Casp3. C Protein expression of cleaved Poly (ADP-ribose) polymerase (c-PARP). D Protein expression of transforming growth factor (TGF)-ß. E Protein expression of Smad3. *n* = 6 for each group. * indicates *p* value  < 0.05; ** indicates *p* value  < 0.01; *** indicates *p* value  < 0.001; **** indicates *p* value  < 0.0001. G1 = (sham-operated control); G2 = sepsis syndrome (SS) + acute kidney ischemia–reperfusion (AKIR); G3 (SS + AKIR + ciprofloxacin administered at 3 h after SS-AKIR induction); G4 [SS + AKIR + iPS-MSCs by intravenous injection at 3 h, after SS-AKIR (i.e., defined as early treatment)]; G5 [SS + AKIR + iPS-MSCs by intravenous injection at 18 h after SS-AKIR (i.e., defined as late treatment) after SS-AKIR induction]; G6 [SS + AKIR + ciprofloxacin and iPS-MSCs at 3 h after SS-AKIR induction]. iPS-MSCs = induced pluripotent stem cells-derived mesenchymal stem cells

The protein expressions of NOX-1, NOX-2 and oxidized protein, three indicators of oxidative stress, and protein expressions of beclin1 and Atg5, two indicators of autophagy, were lowest in group 1, highest in group 2, significantly lower in group 6 than in groups 3 to 5, and significantly lower in group 4 than in groups 3 and 5, but they did not differ between groups 3 and 5 (Fig. 7).

Additionally, the protein expressions of mitochondrial Bax, cleaved caspase 3 and cleaved PARP, three indicators of apoptosis, exhibited an identical pattern of oxidative stress among the groups (Fig. 8). Furthermore, the protein expressions of TGF-ß and Smad3, two indices of fibrosis, also exhibited an identical pattern of oxidative stress among the six groups (Fig. 8).

### Protein expressions of inflammation in kidney by day 5 after SS-AKIR procedure (Fig. 9)

**Fig. 9:**
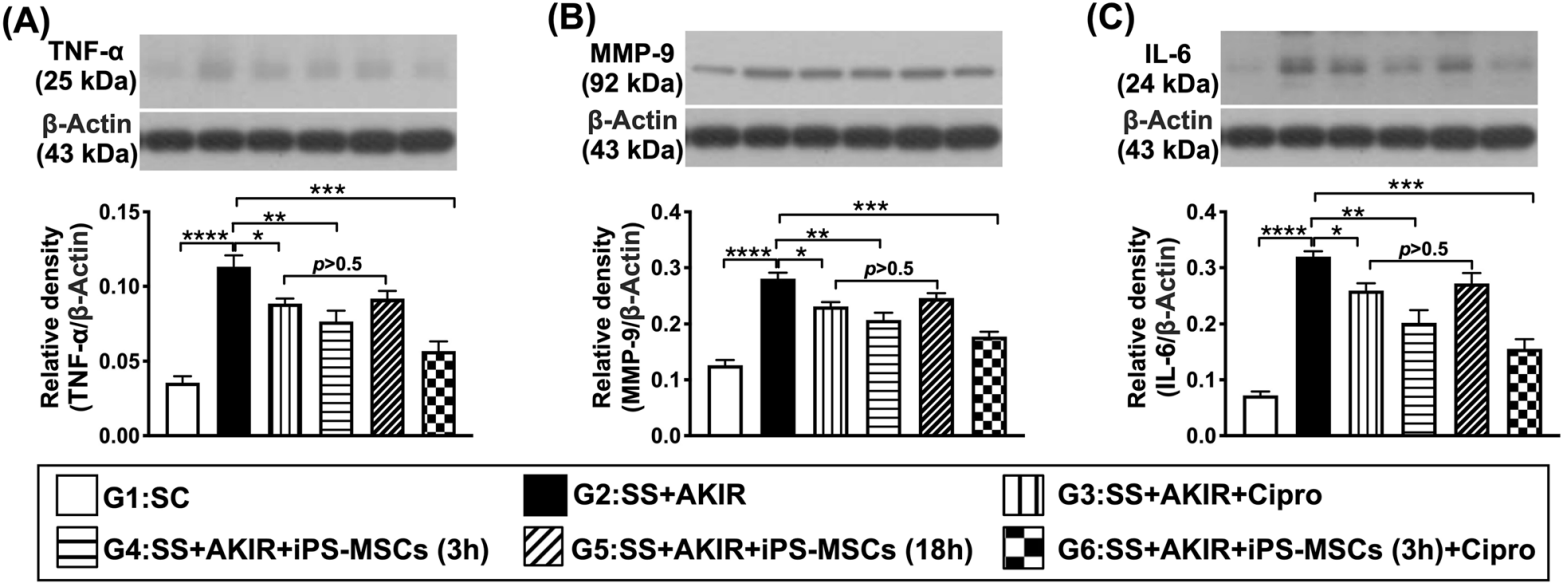
Protein expressions of inflammation in kidney by day 5 after SS-AKIR procedure. A Protein expression of tumor necrosis factor (TNF)-α. B Protein expression of matrix metalloproteinase (MMP)-9. C Protein expression of interleukin (IL)-6. *n* = 6 for each group. * indicates *p* value  < 0.05; ** indicates *p* value  < 0.01; *** indicates *p* value  < 0.001; **** indicates *p* value  < 0.0001. G1 = (sham-operated control); G2 = sepsis syndrome (SS) + acute kidney ischemia–reperfusion (AKIR); G3 (SS + AKIR + ciprofloxacin administered at 3 h after SS-AKIR induction); G4 [SS + AKIR + iPS-MSCs by intravenous injection at 3 h, after SS-AKIR (i.e., defined as early treatment)]; G5 [SS + AKIR + iPS-MSCs by intravenous injection at 18 h after SS-AKIR (i.e., defined as late treatment) after SS-AKIR induction]; G6 [SS + AKIR + ciprofloxacin and iPS-MSCs at 3 h after SS-AKIR induction]. iPS-MSCs = induced pluripotent stem cells-derived mesenchymal stem cells

Protein expressions of TNF-α, MMP-9 and IL-6, three indicators of inflammation, were lowest in group 1, highest in group 2, significantly lower in group 6 than in groups 3 to 5, and significantly lower in group 4 than in groups 3 and 5, but they did not differ between groups 3 and 5.

## Discussion

This study which investigated the therapeutic impact of ciprofloxacin and iPS-MSCs on improving the outcomes in setting of SS-AKIR yielded several clinical striking implications. First, the result of the present study demonstrated that iPS-MSCs were notably comparable to ciprofloxacin on reducing the kidney injury and improving the prognostic outcome. Second, early administration was better than late administration of iPS-MSCs for preservation of integrity of kidney ultrastructure and improvement of prognostic outcome. Third, combined therapy with ciprofloxacin and iPS-MSCs was superior to either one alone in reducing the mortality and protecting the kidney function and architectural integrity against SS-AKIR damage.

Previous studies have clearly demonstrated that the development of AKI in the setting of sepsis caused not only an unacceptable high risk of in-hospital death [25, 26], but also the risk of progression to chronic kidney disease among survivors [27]. One important finding in the present study was that as compared with the SC group, not only the creatinine and BUN levels but also the 5-day mortality were significantly higher in those of SS-AKIR without treatment. Our findings, therefore, supported the findings of the previous studies [25–27]. Another important finding was that iPS-MSCs therapy was comparable with ciprofloxacin on preserving the renal function and kidney ultrastructural integrity as well as on reducing the 5-day mortality. Our previous studies have shown that adipose-derived MSCs were also effective on protecting organ against the SS-induced injury [31, 32, 38, 39]. In this way, our finding corroborated with the finding of our previous studies [31, 32, 38, 39].

Interestingly, our other previous studies have revealed that early administration of stem cells was more advantageous than the late administration of stem cells on protecting the organ from ischemia and hypoxia induced damage [40, 41]. Of importance was that the result of the present study identified that early administration was better than late administration of iPS-MSCs on improving the prognostic outcome. Accordingly, the result of this study was consistent with those from our previous studies [40, 41]. Of particularly important finding was that combined iPS-MSCs and ciprofloxacin was superior to either one alone for protecting the kidney organ and improving the prognostic outcome, highlighting that this strategic management may serve as a therapeutic protection for those severe sepsis patients complicated with major organ failure and are refractory to conventional treatment.

Link between inflammatory and oxidative stress and organ damage along with unfavorable outcomes have been extensively investigated by abundant experimental studies [31–33, 35, 38–41]. An essential finding in the present in vitro study was that the iPS-MSCs had powerful capacity of anti-inflammation and immunomodulation. Another essential fining in the present in vivo study was that as compared with the SC animals, SS-AKIR animals had remarkably increased inflammatory and immunogenic parameters in circulation and spleen as well as inflammatory and oxidative-stress biomarkers in kidney parenchyma. Hence, our findings, in addition to corroborating with the findings of previous studies [31–33, 35, 38–41], not only could explain why the mortality rate was significantly higher but could also explain why the levels of BUN creatinine in circulation and the DNA-damage, apoptotic and fibrotic biomarkers in kidney parenchyma as well as the kidney injury score were substantially increased in SS-AKIR group than in those of control group. Of distinctive finding was that these molecular-cellular perturbations were significantly reversed by iPS-MSCs or ciprofloxacin and furthermore significantly reversed by combined iPS-MSCs and ciprofloxacin treatment.

Our previous study [35] has clearly delineated that IR injury significantly damaged the podocyte components and the integrity of glomerular ultrastructure, resulting in proteinuria and deterioration of renal function. Consistent with the previous study [35], the present study also demonstrated that the integrities of podocyte components (i.e., ZO-1, p-cadherin) and the component of podocyte foot process (i.e., synaptopodin) were significantly reduced, whereas the kidney injury molecule (i.e., KIM-1) was significantly increased in SS-AKIR animals than in those of SC animals. In this way, our findings in addition to strengthening the finding of our previous study [35], could explain why the Ra-Up/Uc was substantially increased SS-AKIR animals. Importantly, these parameters were significantly reversed by iPS-MSCs or ciprofloxacin treatment and further significantly reversed by combined iPS-MSCs and ciprofloxacin treatment.

## Study limitations

This study has limitations. First, the study period was 5 days. Thus, even though the short-term outcomes were attractive and promising, the long-term outcomes from this synergic therapeutic strategy remained uncertain. Second, the sample size in each group was relatively small that could distort the statistical significance when the mortality rate was taken into consideration, resulting in bias that could not be completely ruled out in the present study. Third, there had small sample size among the groups, the statistical significance of mortality could be distorted in the present study.

In conclusion, the results of the present study demonstrated that combined iPS-MSCs and ciprofloxacin treatment provided high-degree collateral benefits on reducing the mortality rate and preserving the functional and ultrastructural integrities of rat kidney in setting of SS-AKIR.

## Supplementary Information

**Additional file 1: Fig. 1.** Schematically illustrate the step-by-step procedure of cell culturing for the iPS derived into iPS-MSCs.

**Additional file 2: Fig. 2.** Illustrating the time courses of differentiation of iPS to iPS-MSCs. iPS = inducible pluripotent stem cell; iPS-MSCs = inducible pluripotent stem cell derived-mesenchymal stem cells.

**Additional file 3: Fig. 3.** Illustrating the iPS-MSC differentiated into adipocytes, chondrocytes, osteoblast. **A**–**C** Illustrating the adipogenic differentiation of iPS-MSCs into adipocytes stained by Oil red O. **D**–**F** Illustrating the chondrogenic differentiation of iPS-MSCs into chondrocytes stained by Alcian Blue. **G**–**I** Illustrating the osteogenic differentiation of iPS-MSCs into osteoblast stained by Alizarin Red S.

## Data Availability

The data that support the findings of this study are available from the corresponding authors upon reasonable request.
