# Supplementary figures and images for: Additional benefit of induced pluripotent stem cell-derived mesenchymal stem cell therapy on sepsis syndrome-associated acute kidney injury in rat treated with antibiotic
Source: Stem Cell Res Ther. 2021 Oct 7;12:526. doi: 10.1186/s13287-021-02582-5 (PMC8499569; doi:10.1186/s13287-021-02582-5)

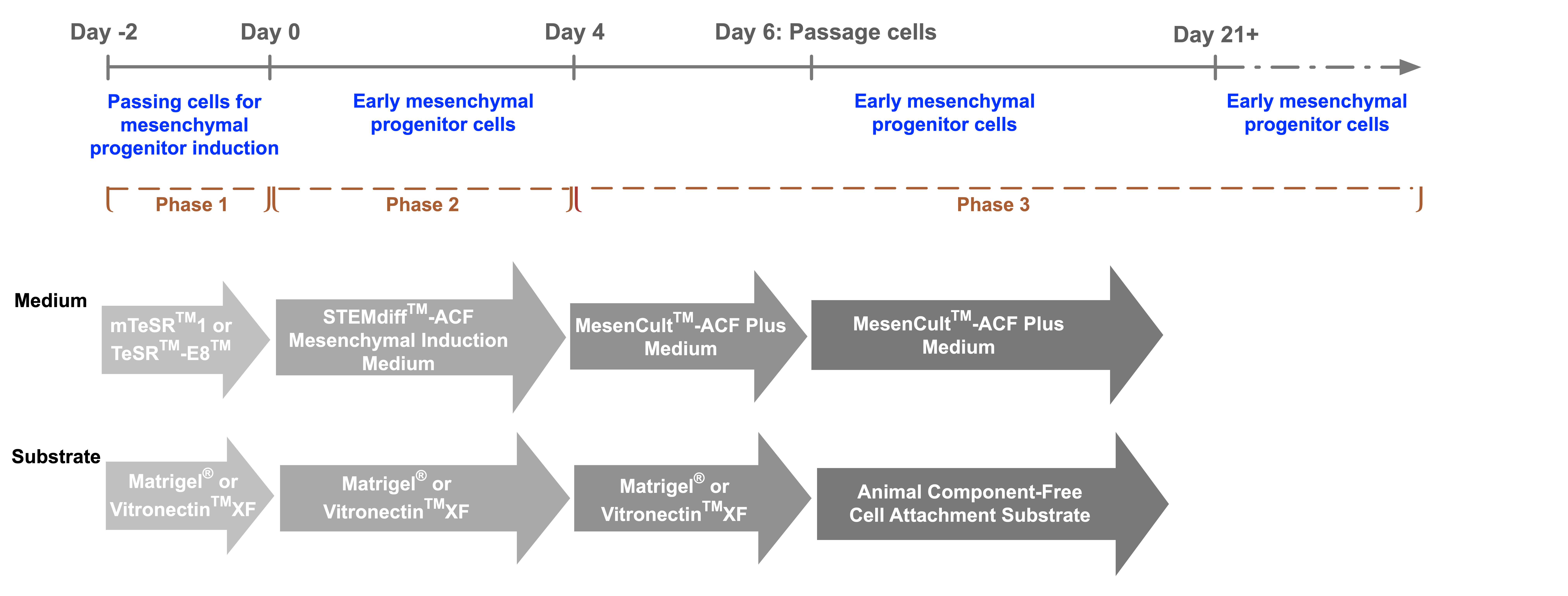

Supplement: Supplementary file 1 — Additional file 1: Fig. 1. Schematically illustrate the step-by-step procedure of cell culturing for the iPS derived into iPS-MSCs. [file 13287_2021_2582_MOESM1_ESM.jpg]

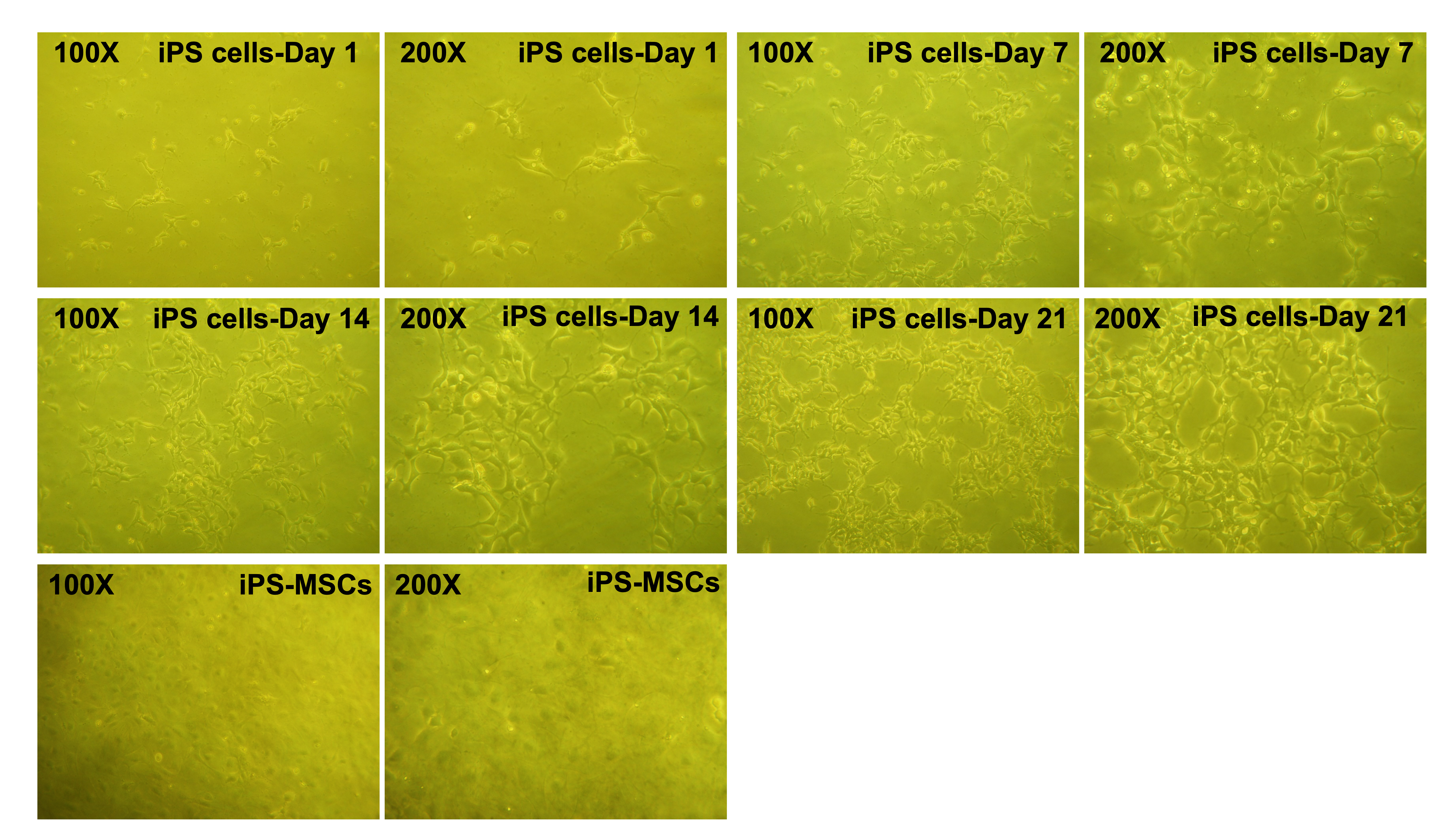

Supplement: Supplementary file 2 — Additional file 2: Fig. 2. Illustrating the time courses of differentiation of iPS to iPS-MSCs. iPS = inducible pluripotent stem cell; iPS-MSCs = inducible pluripotent stem cell derived-mesenchymal stem cells. [file 13287_2021_2582_MOESM2_ESM.jpg]

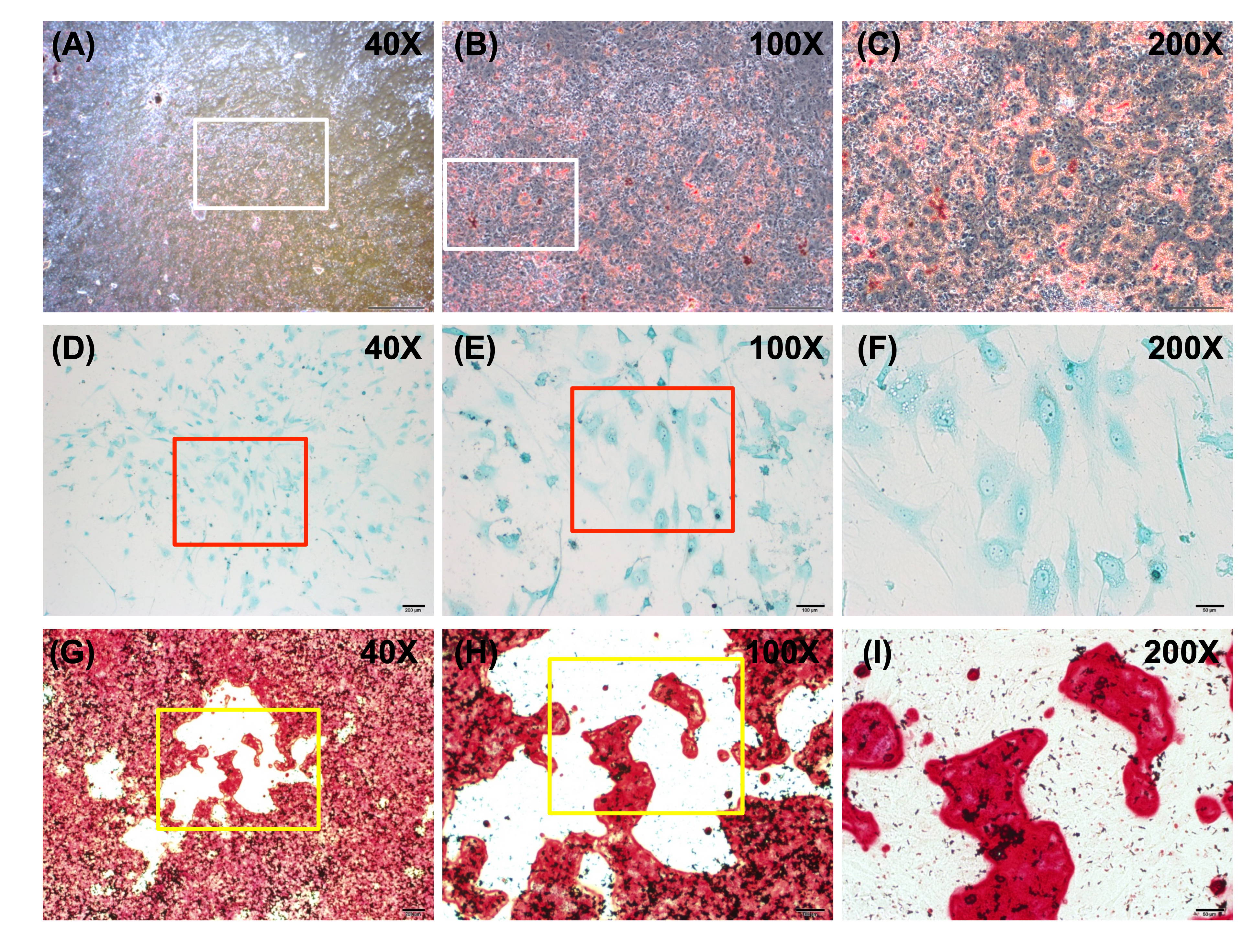

Supplement: Supplementary file 3 — Additional file 3: Fig. 3. Illustrating the iPS-MSC differentiated into adipocytes, chondrocytes, osteoblast. A–C Illustrating the adipogenic differentiation of iPS-MSCs into adipocytes stained by Oil red O. D–F Illustrating the chondrogenic differentiation of iPS-MSCs into chondrocytes stained by Alcian Blue. G–I Illustrating the osteogenic differentiation of iPS-MSCs into osteoblast stained by Alizarin Red S. [file 13287_2021_2582_MOESM3_ESM.jpg]
